# Inhibition of long non-coding RNA XIST upregulates microRNA-149-3p to repress ovarian cancer cell progression

**DOI:** 10.1038/s41419-020-03358-0

**Published:** 2021-02-01

**Authors:** Rong Jiang, Hongyu Zhang, Jinhua Zhou, Juan Wang, Yuejuan Xu, He Zhang, Yanzheng Gu, Fengqing Fu, Yu Shen, Guangbo Zhang, Lanlan Feng, Xueguang Zhang, Youguo Chen, Fangrong Shen

**Affiliations:** 1 Department of Obstetrics and Gynecology, The First Affiliated Hospital of Soochow University, Suzhou, 215000 Jiangsu China; 2 Jiangsu Institute of Clinical Immunology, The First Affiliated Hospital of Soochow University, Suzhou, 215000 Jiangsu China; 3 Jiangsu Key Laboratory of Clinical Immunology, Soochow University, Suzhou, 215000 Jiangsu China; 4 Jiangsu Key Laboratory of Gastrointestinal Tumor Immunology, The First Affiliated Hospital of Soochow University, Suzhou, 215000 Jiangsu China; 5 Department of Gynecology, The Second People’s Hospital of Taizhou, Taizhou, 225500 Jiangsu China

**Keywords:** Cell biology, Diseases

## Abstract

Long non-coding RNAs (lncRNAs) and microRNAs (miRNAs) play critical roles in human diseases. We aimed to clarify the role of lncRNA X-inactive specific transcript (XIST)/miR-149-3p/forkhead box P3 (FOXP3) axis in ovarian cancer (OC) cell growth. XIST, miR-149-3p and FOXP3 expression in OC tissues and cell lines was assessed, and the predictive role of XIST in prognosis of OC patients was analyzed. The OC cell lines were screened and accordingly treated with silenced/overexpressed XIST plasmid or miR-149-3p mimic/inhibitor, and then the proliferation, invasion, migration, colony formation ability, apoptosis, and cell cycle distribution of OC cells were measured. Effect of altered XIST and miR-149-3p on tumor growth in vivo was observed. Online website prediction and dual luciferase reporter gene were implemented to detect the targeting relationship of lncRNA XIST, miR-149-3p, and FOXP3. XIST and FOXP3 were upregulated, whereas miR-149-3p was downregulated in OC tissues and cells. High XIST expression indicated a poor prognosis of OC. Inhibition of XIST or elevation of miR-149-3p repressed proliferation, invasion, migration, and colony formation ability, and promoted apoptosis and cell cycle arrest of HO-8910 cells. In SKOV3 cells upon treatment of overexpressed XIST or reduction of miR-149-3p, there exhibited an opposite tendency. Based on online website prediction, dual luciferase reporter gene, and RNA pull-down assays, we found that there was a negative relationship between XIST and miR-149-3p, and miR-149-3p downregulated FOXP3 expression. This study highlights that knockdown of XIST elevates miR-149-3p expression to suppress malignant behaviors of OC cells, thereby inhibiting OC development.

## Introduction

Ovarian cancer (OC) is the seventh commonest cancer all over the world in women and is the second most prevalent malignant tumor after breast cancer (BC) in women over the age of 40 years, especially in developed countries^1^. OC is a heterogeneous tumor that consists of over 15 tumor types and subtypes based on histological features, and two-thirds of OC cases are diagnosed as high-grade serous types^2^. Despite increased survival rate during the recent decades, two-thirds of patients still die within 10 years of diagnosis. In women diagnosed with advanced-stage invasive epithelial OC, the 5-year survival rate is <20%^3^. Primary cytoreductive surgery followed by platinum-based chemotherapy has been the cornerstone of treatment for OC patients. Despite improvements of the outcomes, current treatments become ineffective after several cycles of management and the recurrence rate is about 80–85%^4^. Thus, it is urgently to explore novel targets for OC treatment.

Long non-coding RNAs (lncRNAs) are a cascade of RNA molecules with transcript lengths over 200 nucleotides and are able to regulate gene expression at epigenetic and transcriptional levels^5^. LncRNAs have been shown to be related with OC development. For instance, lncRNA HOTTIP has been reported to be a crucial indicator of OC prognosis and enhances cell proliferation and invasion^6^, and it has been unearthed that lncRNA FEZF1-AS1 promotes proliferation and inhibits apoptosis in OC^7^. LncRNA X-inactive specific transcript (XIST) is a 17 kb lncRNA that modulates X-chromosome inactivation of mammals to obtain gene dosage equivalence between XX female and XY male^8^. XIST has been considered to promote proliferation and differentiation of OC^9^ and it has also been demonstrated that XIST contributes to progression of cervical cancer^10^. MicroRNAs (miRNAs) are small non-coding RNA molecules able to posttranscriptionally regulate gene expression by targeting the 3′-untranslated region (3′-UTR) of mRNAs^11^. It has been unraveled that some particular miRNAs participate in OC progression, including miR-34^12^ and miR-214^13^. As one of the miRNAs, miR-149 has been identified to suppress the proliferation and increase sensitivity of OC cells to cisplatin^14^, and miR-149-3p has been reported to be a novel biomarker for evaluating the prognosis of patients with epithelial OC and it might have potential therapeutic values^15^. Interestingly, the binding relationship between XIST and miR-149-5p has been confirmed in osteoarthritis^16^, whereas the correlation between miR-149-3p and XIST in OC remains largely unknown. Forkhead box protein P3 (FOXP3) belongs to the forkhead-winged-helix family of transcriptional regulators. Moreover, it has been validated that FOXP3 is able to regulate cell proliferation, migration, and invasion in epithelial OC^17^, and FOXP has also been identified to be associated with poor prognosis in OC^18^. However, the relationship between miR-149-3p and FOXP3 remains to be explored. Therefore, we aimed to verify the role of lncRNA XIST/miR-149-3p/FOXP3 axis in OC progression and we inferred that altered XIST and miR-149-3p may regulate biological functions of OC cells, thereby affecting OC progression.

## Materials and methods

### Ethics statement

Written informed consents were acquired from all patients before this study. The protocol of this study was confirmed by the Ethic Committee of the First Affiliated Hospital of Soochow University. Animal experiments were strictly in accordance with the Guide to the Management and Use of Laboratory Animals issued by the National Institutes of Health. The protocol of animal experiments was approved by the Institutional Animal Care and Use Committee of the First Affiliated Hospital of Soochow University.

### Study subjects

A total number of 45 OC samples were collected from patients (aged 33–74 years, mean age of 52 years), who had accepted resection in the First Affiliated Hospital of Soochow University, and were all confirmed as OC by pathological diagnosis. According to the International Federation of Gynecology and Obstetrics (FIGO) stage^19^, there were 7 cases of I stage, 14 cases of II stage, and 24 cases of III stage. The collected tissues were placed in liquid nitrogen and preserved at −80 °C after 30 min. The patients had not accepted any treatment before the resection. Forty normal ovarian tissues and 22 pairs of non-metastatic lymph nodes and lymph node metastatic tumors were also collected. Complete follow-up data of the patients were obtained through outpatient service and telephone. The follow-up period ended on 30 April 2019 and lasted for 60 months.

### Cell culture and screening

Human normal ovarian epithelial cells (NOECs) and human epithelial OC cells (OVCAR3, CAOV3, SKOV3, HO-8910, and HO-8910PM) were all purchased from Shanghai Institute of Biochemistry and Cell Biology, Chinese Academy of Sciences (Shanghai, China). NOECs were trypsinized for primary culture and placed in keratinocyte serum-free medium containing 100 U/mL penicillin and 100 μg/mL streptomycin for continuous subculture; SKOV3, HO-8910, and HO-8910PM cells were cultured with Roswell Park Memorial Institute (RPMI)-1640 medium containing 100 mL/L fetal bovine serum (FBS); OVCAR3 cells were cultured by RPMI-1640 medium containing 200 mL/L FBS and 155 U/L insulin; CAOV3 cells were cultured with Dulbecco’s modified Eagle medium containing 100 mL/L FBS. All of the above culture solutions contained 100 U/mL penicillin and 100 μg/mL streptomycin. HO-8910 cells had the largest, whereas SKOV3 cells had the least difference in corresponding expression from NOECs; hence, the two OC cell lines were screened for subsequent experiments.

### Cell grouping and transfection

SKOV3 and HO-8910 cells were seeded onto a 24-well plate at 1 × 10^5^ cells/well and then transfected after 80% cell confluence. The transfection was in line with protocols of lipofectamine 2000 reagent (Invitrogen, Inc., CA, USA) and the relative sequences were obtained from GenePharma Co., Ltd (Shanghai, China).

SKOV3 cells were divided into seven groups and respectively transfected with overexpressed (OE)-XIST, miR-149-3p inhibitor, OE-XIST + miR-149-3p mimic, or their negative controls (NCs).

HO-8910 were also classified into seven groups and were respectively transfected with short hairpin RNA (sh)-XIST, miR-149-3p mimic, sh-XIST + miR-149-3p inhibitor, or their NCs.

### Reverse-transcription quantitative PCR

Total RNA in tissues and cells was extracted with RNeasy Plus Mini Kit (QIAGEN company, Hilden, Germany). Reverse transcription was conducted by TaqMan MicroRNA Reverse Transcription Kit (Applied Biosystems, Inc., CA, USA) and the reaction solution was used for fluorescent quantitative PCR (qPCR) based on directions of SYBR Premix Ex Taq^TM^ II kits (Takara Bio, Inc., Otsu, Shiga, Japan) on a BIO-RAD CFX96 instrument. The primers (Table 1) were designed by Shanghai Sangon Biotechnology Co., Ltd (Shanghai, China). U6 was used as the loading control of miR-149-3p and glyceraldehyde phosphate dehydrogenase (GAPDH) was used as the loading control of XIST and FOXP3. The data were analyzed by 2^−△△Ct^ method.

**Table 1:** Primer sequence.

Gene	Primer sequence (5′-3′)
*XIST*	Forward: CGGGTCTCTTCAAGGACATTTAGCC
	Reverse: GCACCAATACAGAGGAATGGAGGG
*MiR-149-3p*	Forward: ACACTCCAGCTGGGAGGGAGGGACGGGGGC
	Reverse: CTCAACTGGTGTCGTGGA
*FOXP3*	Forward: ATCGCTGCTAGCTACTTAGCTA
	Reverse: CTGATCGTGAACTGCCGTGGCTA
*GAPDH*	Forward: GAAGATGGTGATGGGATTTC
	Reverse: GAAGGTGAAGGTCGGAGT
*U6*	Forward: CTCGCTTCGGCAGCACA
	Reverse: AACGCTTCACGAATTTGCGT

*FOXP3* forkhead box P3, *GAPDH* glyceraldehyde phosphate dehydrogenase, *miR-149-3p* microRNA-149-3p, *XIST* X-inactive specific transcript.

### Western blot analysis

Total protein was extracted from OC tissues and cells and the protein concentration was measured. The proteins were conducted with 10% SDS-polyacrylamide gel electrophoresis and transferred onto membranes, which were then blocked with 5% skim milk powder at 4 °C overnight. Subsequently, the membranes were probed with primary antibody against FOXP3 (1 : 1000, Invitrogen, Inc.) and GAPDH (1 : 1000, Santa Cruz Biotechnology, Inc., CA, USA) overnight, and then were re-probed with horseradish peroxidase-conjugated immunoglobulin G (1 : 1000, BOSTER Biological Technology Co., Ltd, Wuhan, Hubei, China) secondary antibody at 37 °C for 1 h, followed by soaked in enhanced chemiluminescent reaction solution (Pierce, Rockford, IL, USA) for 1 min. With the fluid removed, the membranes were covered by food wrap and exposed in the dark, then developed, fixed, and observed. GAPDH was taken as the internal reference and the protein bands were analyzed using ImageJ2x software.

### MTT assay

Cells in each group were trypsinized, seeded, and cultured for 0, 12, 24, 36, and 48 h, respectively. The cell viability was performed with MTT (3-(4,5-dimethyl-2-thiazolyl)-2,5-diphenyl-2-H-tetrazolium bromide) assay as previously described^20^. The optical density at 490 nm of each well was analyzed by a microplate reader (Anthos Labtec, Wals, Austria).

### Colony formation assay

Cells in each group that had been cultured for 24 h were trypsinized and seeded onto 35 mm culture dishes for 10-day culture and the medium was changed every 3 days. After this, the cells were fixed with 40 g L^−1^ paraformaldehyde and stained by 1 g L^−1^ crystal violet dye solution for 20 min. The number of colonies (>50 cells) was counted under a microscope.

### Flow cytometry

#### Determination of cell cycle distribution

SKOV3 and HO-8910 cells that had been transfected for 48 h were trypsinized and resuspended by phosphate-buffered saline (PBS). The cell suspension was centrifuged at 2000 r.p.m. for 5 min with the supernatant discarded and there was a backflow of 50 μL remaining PBS. The cells were resuspended by flicking the tubes and appended with 2 mL PBS, and then centrifuged at 2000 r.p.m. for 5 min with the supernatant removed. The cell concentration was adjusted to 1 × 10^6^ cells/mL by adding 1 × binding buffer and the cells were added with 1 mL PBS, 20 μL propidium iodide (PI), 5 μL RNase, and 1 μL 20% Nonidet P40, then transferred into the flow tubes. Each sample was added with 300–500 μL labeling liquid for 3–5 min and the cell cycle distribution was determined.

#### Detection of apoptosis

SKOV3 and HO-8910 cells that had been transfected for 48 h were centrifuged and transferred into 2.0 mL Eppendorf tubes, which were fixed with 1 mL absolute ethanol at −20 °C for over 24 h. After centrifugation, the cells were resuspended with 1 mL 5% PBS and rinsed again. The sediment was suspended with 80 μL 1 mg/mL Rnase A and placed at 37 °C for 30 min, then appended with 400 μL 50 μg/mL PI in dark for 10 min. The apoptosis of SKOV3 and HO-8910 cells was measured.

### Dual luciferase reporter gene assay

A biological online prediction website (https://cm.jefferson.edu/rna22/Precomputed/) was used to predict the potential binding sites of miR-149-3p and XIST. XIST sequence was obtained and the sequence segments was synthetized by PCR amplification with genomic DNA as the template. The segment was digested and purified, then connected with pGL3-Control vector by T4 ligase and transformed. After the identification of positive colony, the sample was seeded into Luria-Bertani medium containing ampicillin and incubated at 37 °C for 12–16 h. Afterwards, agarose gel electrophoresis, sequencing, and comparative analysis were successively performed. MiR-149-3p-binding site mutant (MUT) vector of pGL3-XIST was synthetized by Wuhan GeneCreate Biological Engineering Co., Ltd (Hubei, China). Wild-type (WT) plasmid containing target sequence was named as XIST-WT and MUT type plasmid that had undergone site-directed mutagenesis was defined as XIST-MUT. SKOV3 and HO-8910 cells were detached, seeded, and incubated until cell confluence reached 60%. The transfection was in line with instructions of X-tremegene HP transfection reagent (Roche, Ltd, Basel, Switzerland): transfection reagent and XIST-WT or XIST-MUT with miR-150-3p mimic, or its NC were added into opti-minimum essential medium for 20 min, and then appended onto cell culture plates and incubated for 6 h. Each well was supplemented with 200 μL complete medium containing 10% FBS. The medium was removed after 48 h incubation and the cells were reacted with 300 μL 1 × passive lysis buffer at 4 °C for 20 min. Subsequently, 40 μL cell lysis buffer was appended on the Lockwell maxisorp plates and added with 20 μL luciferase assay reagent, followed by detection of firefly luciferase activity by a microplate reader. Fifty microliters of Stop&Glo^®^ reagent was supplemented into each well and the *Renilla* luciferase activity was gauged by a microplate reader. Finally, the relative luciferase activity of each sample was calculated and data were analyzed.

A bioinformatic online website (https://cm.jefferson.edu/rna22/Precomputed/) was utilized to predict the target relation between miR-149-3p and FOXP3, and binding sites of miR-149-3p and FOXP3 3′-UTR. FOXP3 3′-UTR promoter sequence containing binding sites of miR-149-3p was synthetized. FOXP3 3′-UTR WT plasmid (FOXP3-WT) and FOXP3 3′-UTR MUT plasmid (FOXP3-MUT) were constructed by Wuhan GeneCreate Biological Engineering. SKOV3 and HO-8910 cells were detached, seeded, cultured, and transfected. The luciferase activity of each sample was determined and the relative luciferase activity was calculated as luciferase activity rate of *Renilla*/firefly.

### Subcutaneous tumorigenesis in nude mice

Forty-two male nude mice aged 4–6 weeks old (Experimental Animal Center, Yunnan University, Yunan, China) were randomly classified into 14 groups and were subcutaneously injected with 0.1 mL OC cell suspension according to the cell grouping. Three mice in each group were placed in the same breeding box and kept in an specific pathogen free (SPF) animal laboratory. Nude mice need to adapt to the environment in an SPF animal laboratory for a week, during which time they should change their feed, bedding, and water bottles, and their health status were observed daily. One week later, each group of cultured OC cells were made into cell suspension and placed in cell cryotubes, and 0.1 mL of cell suspension (1 × 10^6^) per mouse was injected under the skin of the back of the neck. Before injection, 75% alcohol should be used to disinfect the injection site. After injection, the injection site was pressed with a sterile cotton swab to prevent cell suspension from flowing out of the needle well. The tumor growth was observed after 3–5 days of the injection. Nude mice were normally weighed and tumor volume was measured. On the day 25 of injection, the nude mice were killed and the xenografts were collected and weighed.

### Statistical analysis

All data analyses were conducted using SPSS 21.0 software (IBM, Corp., Armonk, NY, USA). The measurement data were expressed as mean ± SD. The *t*-test was performed for comparisons between two groups, one-way analysis of variance (ANOVA) was used for comparisons among multiple groups and Tukey’s post hoc test was used for pairwise comparisons after one-way ANOVA. Kaplan–Meier was used to analyze the prognosis of OC patients. *P*-value < 0.05 was indicative of statistically significant difference.

## Results

### XIST and FOXP3 are upregulated, whereas miR-1**4**9-3p is downregulated in OC tissues, and high expression of XIST indicates a poor prognosis of OC patients

**Fig. 1:**
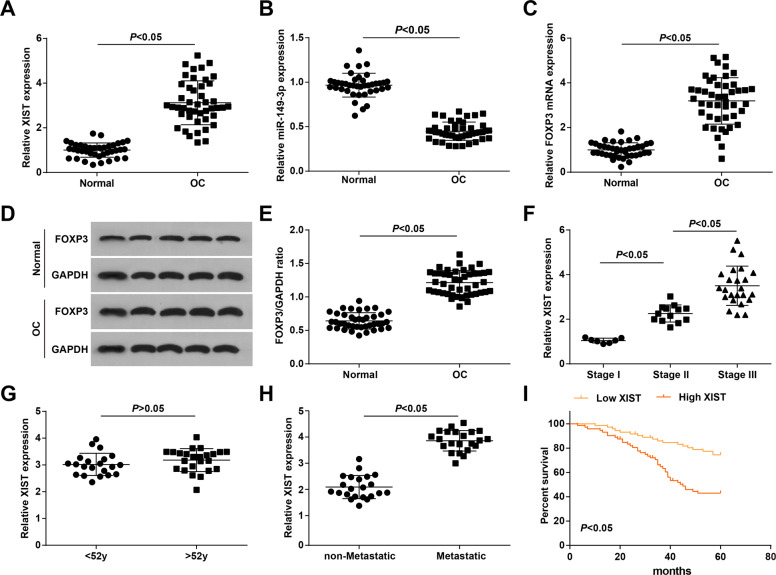
XIST and FOXP3 are upregulated, whereas miR-149-3p is downregulated in OC tissues, and high expression of XIST indicates a poor prognosis of OC patients. **A** XIST expression in normal ovarian tissues and OC tissues. **B** miR-149-3p expression in normal ovarian tissues and OC tissues. **C** FOXP3 mRNA expression in normal ovarian tissues and OC tissues. **D** Protein band of FOXP3 in normal ovarian tissues and OC tissues. **E** Protein expression of FOXP3 in normal ovarian tissues and OC tissues. **F** Impact of FIGO stage on XIST expression. **G** Impact of age on XIST expression. **H** Impact of LNM on XIST expression. **I** Predictive role of XIST in prognosis of OC patients. The measurement data were expressed as mean ± SD, *t*-test was performed for comparisons between two groups, and Kaplan–Meier analysis was used to analyze the prognosis of OC patients.

Levels of XIST, miR-149-3p, and FOXP3 were determined by reverse-transcription qPCR (RT-qPCR) and western blot assay, and we found that (Fig. 1A–E) XIST and FOXP3 expression was upregulated, whereas miR-149-3p expression was downregulated in OC tissues vs. those in normal ovarian tissues.

OC patients were grouped based on FIGO stage and XIST expression in patients of different stages was evaluated by RT-qPCR. It came out that (Fig. 1F, G) XIST expression in patients at advanced stages was higher than those at early stages, whereas there showed no noticeable difference in XIST expression among patients of difference ages. The detection results of XIST expression in 22 pairs of non-metastatic lymph nodes and metastatic lymph nodes showed that in comparison to non-metastatic lymph nodes, XIST expression was elevated in metastatic lymph nodes (Fig. 1H). Based on the median of relative expression of XIST, OC patients were divided into high and low expression groups, and the predictive role of XIST in prognosis of OC patients was evaluated by Kaplan–Meier analysis, and the findings (Fig. 1I) suggested that high XIST expression was associated with a poor prognosis of OC.

### Expression and transfection efficiency identification of XIST and FOXP3 in OC cell lines

**Fig. 2:**
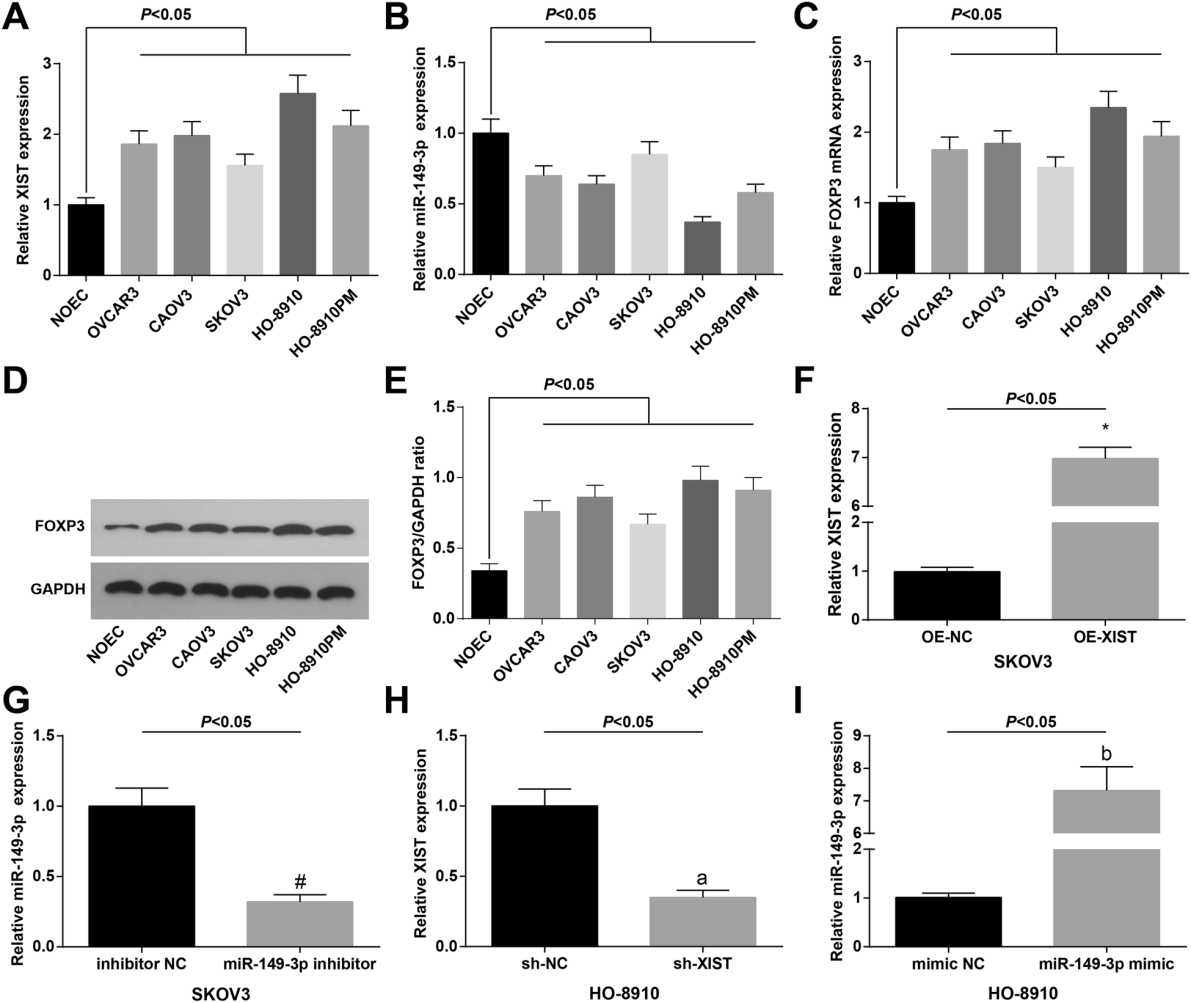
XIST and FOXP3 are upregulated, whereas miR-149-3p is downregulated in OC cells. **A** RT-qPCR detection of XIST expression in OC cell lines (OVCAR3, CAOV3, SKOV3, HO-8910, and HO-8910PM) and human normal ovarian cell line (NOEC). **B** RT-qPCR detection of miR-149-3p expression in OC cell lines (OVCAR3, CAOV3, SKOV3, HO-8910, and HO-8910PM) and human normal ovarian cell line (NOEC). **C** RT-qPCR detection of FOXP3 mRNA expression in OC cell lines (OVCAR3, CAOV3, SKOV3, HO-8910, and HO-8910PM) and human normal ovarian cell line (NOEC). **D** Protein bands of FOXP3 in OC cell lines (OVCAR3, CAOV3, SKOV3, HO-8910, and HO-8910PM) and human normal ovarian cell line (NOEC). **E** Western blot assay detection of FOXP3 protein expression in OC cell lines (OVCAR3, CAOV3, SKOV3, HO-8910, and HO-8910PM) and human normal ovarian cell line (NOEC). **F** XIST expression in SKOV3 cells after XIST overexpression detected using RT-qPCR. **G** miR-149-3p expression in SKOV3 cells after miR-149-3p inhibition detected using RT-qPCR. **H** XIST expression in HO-8910 cells after XIST knockdown detected using RT-qPCR. **I** miR-149-3p expression in HO-8910 cells after miR-149-3p elevation detected using RT-qPCR. The measurement data were expressed as mean ± SD, one-way ANOVA was used for comparisons among multiple groups, and Tukey’s post hoc test was used for pairwise comparisons after one-way ANOVA.

To explore the effect of XIST and miR-146-3p on XIST, miR-149-3p, and FOXP3 expression in SKOV3 and HO-8910 cells, XIST, miR-149-3p, and FOXP3 expression in OC cell lines and NOECs were determined by RT-qPCR and western blot assay, and it was observed that (Fig. 2A–E) OC cell lines had higher XIST and FOXP3 expression, and lower miR-149-3p expression vs. NOECs. Among the OC cell lines, SKOV3 cells had the least, whereas HO-8910 cells had the largest difference in XIST and FOXP3 expression from NOECs.

SKOV3 cells were transfected with OE-XIST, miR-149-3p inhibitor, OE-XIST + miR-149-3p mimic, or their NCs, and HO-8910 were transfected with sh-XIST, miR-149-3p mimic, sh-XIST + miR-149-3p inhibitor, or their NCs, to investigate the effects of XIST and miR-149-3p on OC cells. RT-qPCR was used to assess the transfection efficiency. The results suggested that in SKOV3 cells (Fig. 2F, G), transfection of OE-XIST and the transfection of miR-149-3p inhibitor inhibited miR-149-3p expression; in HO-8910 cells (Fig. 2H, I), treatment of sh-XIST reduced XIST expression and treatment of miR-149-3p mimic upregulated miR-149-3p.

### Inhibited XIST or elevated miR-149-3p represses proliferation and promotes apoptosis of OC cells, and arrests OC cells at G0/G1 p**h**ase

**Fig. 3:**
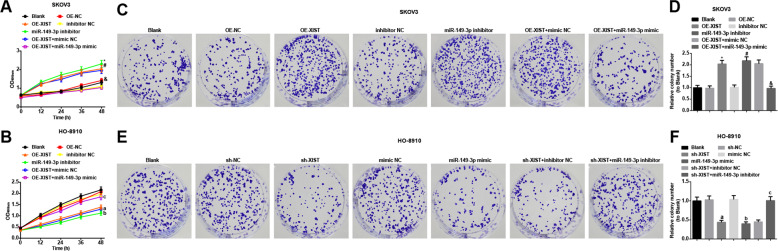
Inhibited XIST or elevated miR-149-3p represses proliferation and colony formation ability of OC cells. **A** Viability of SKOV3 cells in each group detected by MTT assay. **B** Viability of HO-8910 cells in each group detected by MTT assay. **C** Colony formation images of SKOV3 cells in each group. **D** Number of colonies of SKOV3 cells in each group. **E** Colony formation images of HO-8910 cells in each group. **F** Number of colonies of HO-8910 cells in each group; *P < 0.05 vs. the OE-NC group, ^#^*P* < 0.05 vs. the inhibitor NC group, & *P* < 0.05 vs. the OE-XIST + mimic NC group; in HO-8910 cells, a *P* < 0.05 vs. the sh-NC group, b *P* < 0.05 vs. the mimic NC group, c *P* < 0.05 vs. the sh-XIST + inhibitor NC group; the measurement data were expressed as mean ± SD, one-way ANOVA was used for comparisons among multiple groups and Tukey’s post hoc test was used for pairwise comparisons after one-way ANOVA.

Proliferation and colony formation ability of OC cells were determined by MTT and colony formation assay. The results indicated that in SKOV3 cells (Fig. 3A, C, D), proliferation and colony formation ability of cells were promoted by treatment of OE-XIST or miR-149-3p inhibitor; the effect of OE-XIST on proliferation and colony formation ability of SKOV3 cells was reversed by miR-149-3p mimic.

In HO-8910 cells (Fig. 3B, E, F), proliferation and colony formation ability of cells were repressed by treatment of sh-XIST or miR-149-3p mimic; miR-149-3p inhibitor abolished the impact of sh-XIST on proliferation and colony formation ability of HO-8910 cells.

**Fig. 4:**
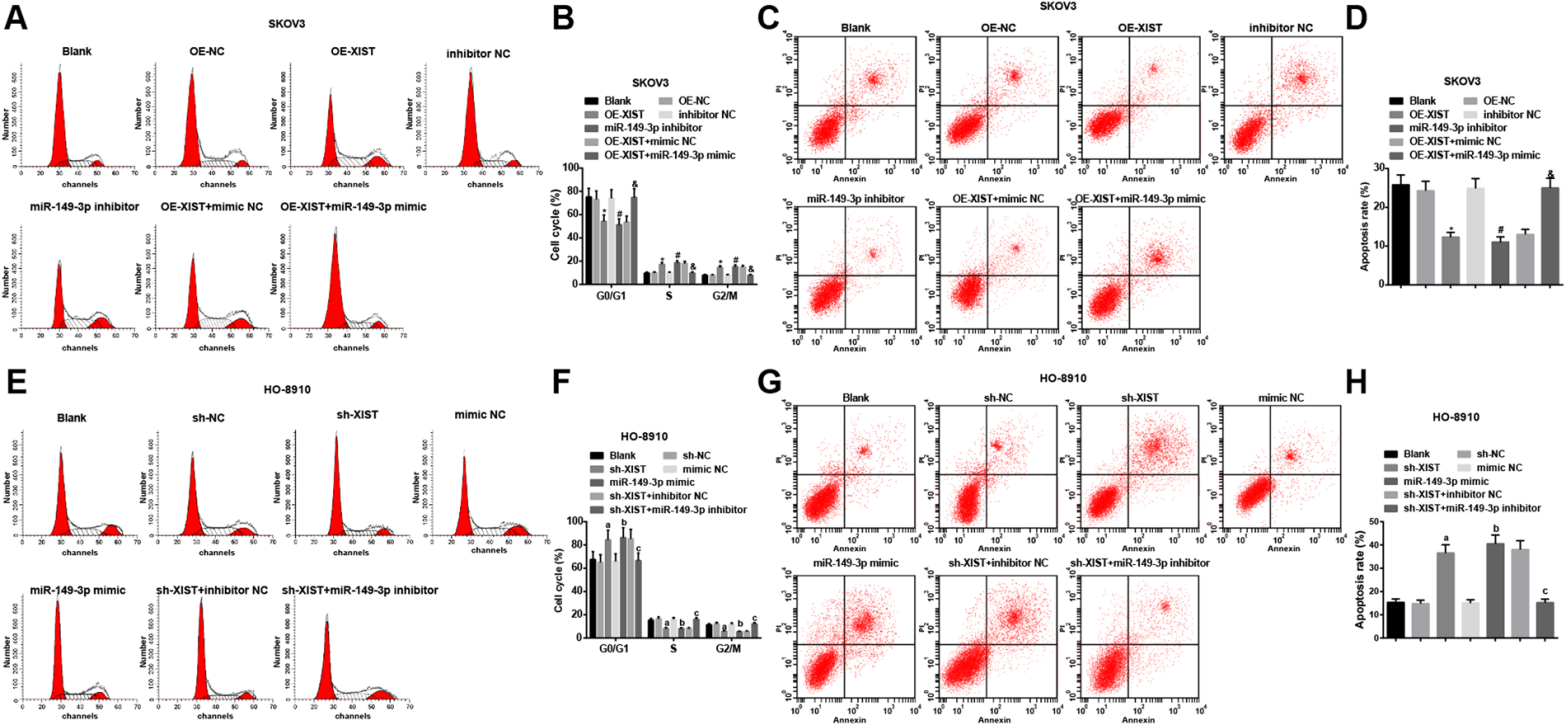
Inhibited XIST or elevated miR-149-3p promotes apoptosis of OC cells, and arrests OC cells at G0/G1 phase. **A** Cell cycle distribution of SKOV3 cells was determined by flow cytometry. **B** SKOV3 cell cycle arrest. **C** SKOV3 cell apoptosis was detected by flow cytometry. **D** Apoptosis rate of SKOV3 cells in each group. **E** Cell cycle distribution of HO-8910 cells was determined by flow cytometry. **F** HO-8910 cell cycle arrest. **G** HO-8910 cell apoptosis was detected by flow cytometry. **H** Apoptosis rate of HO-8910 cells in each group. In SKOV3 cells, *P < 0.05 vs. the OE-NC group, ^#^*P* < 0.05 vs. the inhibitor NC group, &*P* < 0.05 vs. the OE-XIST + mimic NC group; in HO-8910 cells, a *P* < 0.05 vs. the sh-NC group, b *P* < 0.05 vs. the mimic NC group, c *P* < 0.05 vs. the sh-XIST + inhibitor NC group; the measurement data were expressed as mean ± SD, one-way ANOVA was used for comparisons among multiple groups, and Tukey’s post hoc test was used for pairwise comparisons after one-way ANOVA.

Apoptosis and cell cycle distribution were measured by flow cytometry and we found that in SKOV3 cells (Fig. 4A–D), OE-XIST or miR-149-3p inhibitor arrested cells in S and G2/M phases, and reduced the apoptosis rate of SKOV3 cells; the effects of OE-XIST on cell cycle arrest and apoptosis rate of SKOV3 cells were reversed by miR-149-3p mimic.

In HO-8910 cells (Fig. 4E–H), sh-XIST or miR-149-3p mimic arrested cells in G0/G1 phase and augmented apoptosis rate HO-8910 cells; the effects of sh-XIST on cell cycle arrest and apoptosis rate of HO-8910 cells were reversed by miR-149-3p inhibitor.

### Inhibited XIST or elevated miR-149-**3**p suppresses migration and invasion of OC cells

**Fig. 5:**
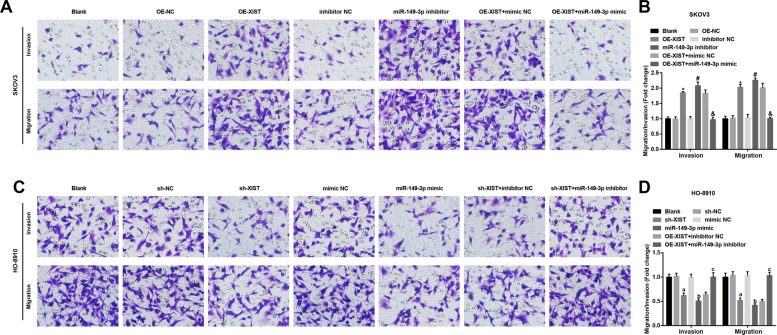
Inhibited XIST or elevated miR-149-3p suppresses migration and invasion of OC cells. A, invasion and migration of SKOV3 cells were determined by Transwell assay; B, numbers of invasive and migratory SKOV3 cells in each group; C, invasion and migration of HO-8910 cells were determined by Transwell assay; D, numbers of invasive and migratory HO-8910 cells in each group; in SKOV3 cells, *P < 0.05 *vs* the OE-NC group, ^#^*P* < 0.05 *vs* the inhibitor NC group, & *P* < 0.05 *vs* the OE-XIST + mimic NC group; in HO-8910 cells, a *P* < 0.05 *vs* the sh-NC group, b *P* < 0.05 *vs* the mimic NC group, c *P* < 0.05 *vs* the sh-XIST + inhibitor NC group; the measurement data were expressed as mean ± standard deviation, one-way ANOVA was used for comparisons among multiple groups and Tukey’s post hoc test was used for pairwise comparisons after one-way ANOVA.

Invasion and migration abilities of OC cells were measured by Transwell assay and we found that in SKOV3 cells (Fig. 5A, B), the invasive and migratory cells were increased by OE-XIST or miR-149-3p inhibitor; miR-149-3p mimic abrogated the impact of OE-XIST on invasion and migration abilities of SKOV3 cells.

In HO-8910 cells (Fig. 5C, D), the invasive and migratory cells were decreased by sh-XIST or miR-149-3p mimic; effects of sh-XIST on invasion and migration abilities of HO-8910 cells were abolished by miR-149-3p inhibitor.

### There is a negative relationship bet**w**een miR-149-3p and XIST

**Fig. 6:**
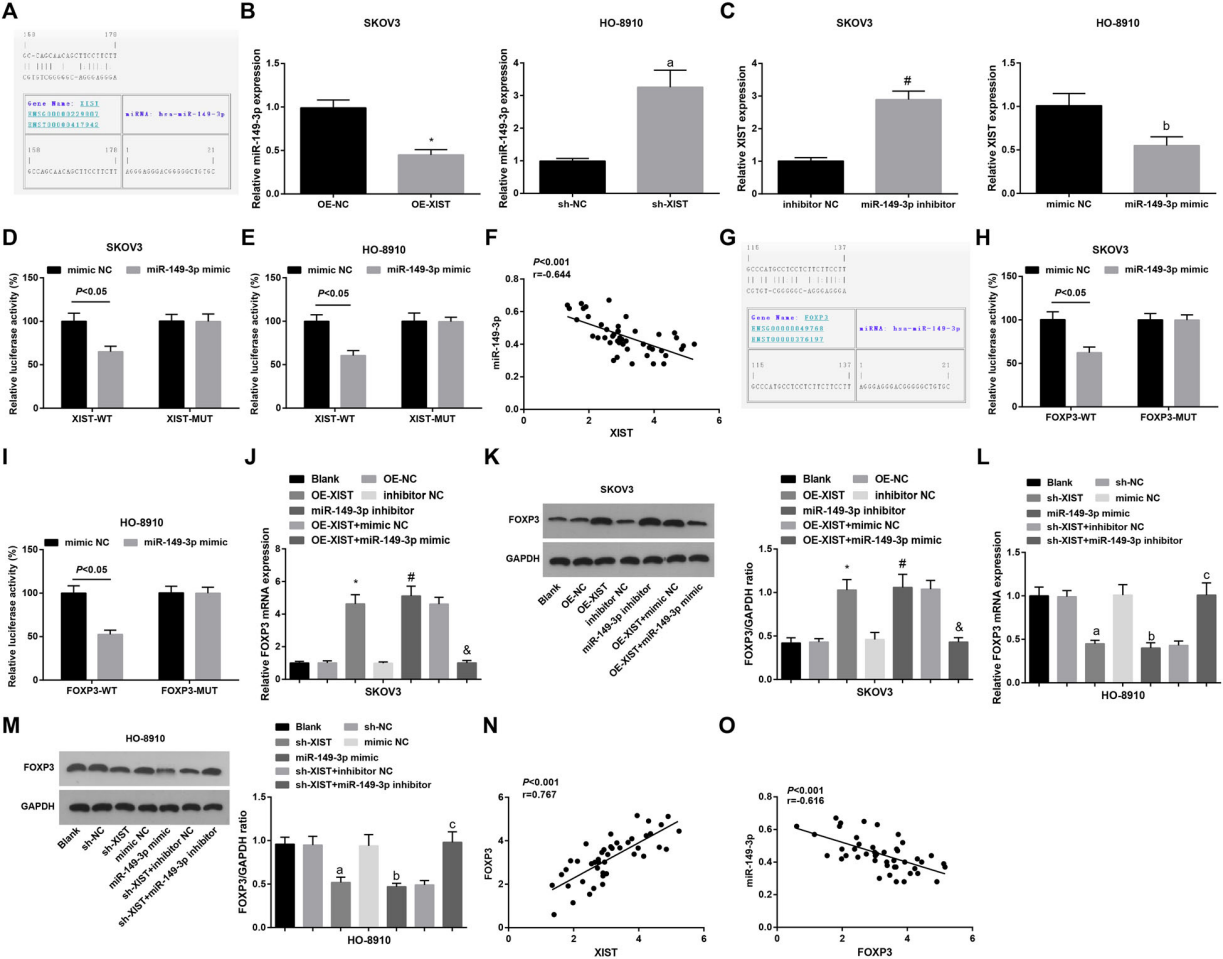
MiR-149-3p particularly binds to XIST and FOXP3 is the target gene of miR-149-3p. A, binding sites of XIST and miR-149-3p were predicted by an online website; B, expression of miR-149-3p after XIST was up- or downregulated detected using RT-qPCR; C, expression of XIST after miR-149-3p was up- or downregulated detected using RT-qPCR; D&E, regulatory relation between XIST and miR-149-3p in SKOV3 and HO-8910 cells was confirmed by dual luciferase reporter gene assay; F, correlation between expression of XIST and miR-149-3p analyzed using Pearson test; G, binding sites between miR-149-3p and FOXP3 predicted at a bioinformatic website; H&I, regulatory relationship between miR-149-3p and FOXP3 in SKOV3 and HO-8910 cells confirmed using dual luciferase reporter gene assay; J, FOXP3 mRNA expression in SKOV3 cells assessed using RT-qPCR; K, FOXP3 protein expression in SKOV3 cells assessed using Western blot analysis; L, FOXP3 mRNA expression in HO-8910 cells assessed using RT-qPCR; M, FOXP3 protein expression in HO-8910 cells assessed using Western blot analysis; N, correlation between FOXP3 and XIST analyzed using Pearson test; O, correlation between FOXP3 and miR-149-3p analyzed using Pearson test; in SKOV3 cells, *P < 0.05 *vs* the OE-NC group, ^#^*P* < 0.05 *vs* the inhibitor NC group, & *P* < 0.05 *vs* the OE-XIST + mimic NC group; in HO-8910 cells, a *P* < 0.05 *vs* the sh-NC group, b *P* < 0.05 *vs* the mimic NC group, c *P* < 0.05 *vs* the sh-XIST + inhibitor NC group; the measurement data were expressed as mean ± standard deviation, *t*-test was performed for comparisons between two groups, one-way ANOVA was used for comparisons among multiple groups and Tukey’s post hoc test was used for pairwise comparisons after one-way ANOVA.

LncRNAs are known to function through their interaction with downstream target miRNAs^21^. To identify whether XIST could interact with miRNA to regulate the OC cell proliferation and tumor growth, we searched for the bioinformatic website (https://cm.jefferson.edu/rna22/) to predict that there existed binding sites between XIST and miR-149-3p (Fig. 6A). The expression of miR-149-3p in SKOV3 and HO-8910 cells was assessed using RT-qPCR after XIST was knocked down or upregulated, and we found that in SKOV3 cells, XIST overexpression inhibited miR-149-3p expression; in HO-8910 cells, XIST reduction increased miR-149-3p expression (Fig. 6B). Then, we regulated miR-149-3p expression to verify whether miR-149-3p could affect XIST expression. As shown in Fig. 6C, in SKOV3 cells, XIST was OE after miR-149-3p was downregulated; in HO-8910 cells, XIST was reduced after miR-149-3p was upregulated. The dual luciferase reporter gene assay was performed to further confirm the direct binding relationship between XIST and miR-149-3p. The results revealed that in SKOV3 and HO-8910 cells, the co-transfection of FOXP3-WT and miR-149-3p mimic decreased the luciferase activity, which was not affected by the co-transfection of FOXP3-MUT and miR-149-3p mimic (Fig. 6D, E). To explore whether there existed a negative relationship between XIST and miR-149-3p, RT-qPCR was conducted in 45 OC tissues. The results indicated that the expression levels of XIST and miR-149-3p were in a negative correlation (*r* = −0.644, *P* < 0.001, Fig. 6F). These data suggested that XIST and miR-149-3p negatively regulated each other in OC.

FOXP3 is a forkhead transcription factor and miRNAs can directly bind to FOXP3 3′-UTR and inhibit its expression^22^. First, a bioinformatic website confirmed the potential binding sites of miR-149-3p in FOXP3 3′-UTR (Fig. 6G). The results of dual luciferase reporter gene assay showed that (Fig. 6H, I) after SKOV3 and HO-8910 cells were co-transfected with FOXP3-WT and miR-149-3p mimic, the relative luciferase activity of SKOV3 and HO-8910 cells was decreased significantly, whereas the co-transfection of FOXP3-MUT and miR-149-3p did not affect the relative luciferase activity of SKOV3 and HO-8910 cells.

In addition, RT-qPCR and western blot analysis showed that overexpression of XIST or inhibition of miR-149-3p significantly increased FOXP3 mRNA and protein expression in SKOV3 cells, and the upregulation of miR-149-3p could reduce OE-XIST-induced FOXP3 expression (Fig. 6J, K); in HO-8910 cells, the knockout of XIST or elevation of miR-149-3p significantly decreased FOXP3 mRNA and protein expression, whereas miR-149-3p inhibitor elevated the sh-XIST-induced FOXP3 mRNA and protein expression (Fig. 6L, M). Finally, Pearson’s test showed that FOXP3 was positively correlated with XIST (*r* = 0.767, *P* < 0.001, Fig. 6N) and FOXP3 was negatively correlated with miR-149-3p (*r* = −0.616, *P* < 0.001, Fig. 6O). These results indicated that FOXP3 is a direct target gene of miR-149-3p and XIST can regulate FOXP3 in OC cells by regulating miR-149-3p.

### Inhibited XIST or elevated miR-**1**49-3p restricts ovarian tumor growth in vivo

**Fig. 7:**
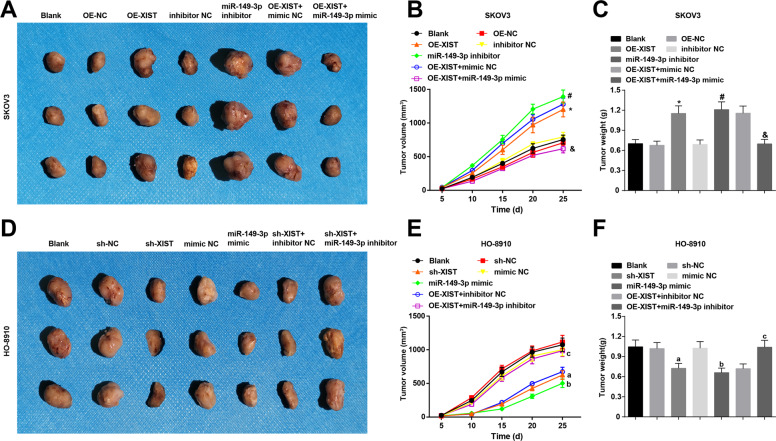
Inhibited XIST or elevated miR-149-3p restricts ovarian tumor growth in vivo. A, figures for SKOV3 xenografts 25 day later in each group; B, growth curve for the volume of SKOV3 xenografts in each group; C, weight of SKOV3 xenografts 25 day later in each group; D, figures for HO-8910 xenografts 25 day later in each group; E, growth curve for the volume of HO-8910 xenografts in each group; F, weight of HO-8910 xenografts 25 day later in each group; in SKOV3 cells, *P < 0.05 *vs* the OE-NC group, ^#^*P* < 0.05 *vs* the inhibitor NC group, & *P* < 0.05 *vs* the OE-XIST + mimic NC group; in HO-8910 cells, a *P* < 0.05 *vs* the sh-NC group, b *P* < 0.05 *vs* the mimic NC group, c *P* < 0.05 *vs* the sh-XIST + inhibitor NC group; the measurement data were expressed as mean ± standard deviation, one-way ANOVA was used for comparisons among multiple groups and Tukey’s post hoc test was used for pairwise comparisons after one-way ANOVA.

Results of subcutaneous tumorigenesis in nude mice mirrored that in SKOV3 xenografts (Fig. 7A–C), tumor weight and volume were both increased by OE-XIST or miR-149-3p inhibitor; effect of OE-XIST on tumor weight and volume was reversed by miR-149-3p mimic.

In HO-8910 xenografts (Fig. 7D–F), tumor weight and volume were reduced by sh-XIST or miR-149-3p mimic; impacts of sh-XIST on tumor weight and volume were abrogated by miR-149-3p inhibitor.

## Discussion

Among all types of malignancies, OC is a leading cause of cancer-correlated death in women and the most fatal gynecologic cancer^1^. According to the competing endogenous RNA hypothesis (also known as the sponge function), lncRNAs are capable of regulating gene expression through titrating miRNAs to participate in various biological processes^23^. We focused on the role of lncRNA XIST/miR-149-3p/FOXP3 axis in OC progression and we found that inhibition of lncRNA XIST could bound with miR-149-3p to repress malignant episodes of OC cells.

We determined the expression levels of XIST, miR-149-3p, and FOXP3 in OC tissues and cells. The results implied that XIST and FOXP3 were upregulated, whereas miR-149-3p was downregulated in both OC tissues and cell lines, respectively, compared with normal ovarian tissues and NOECs. Consistently, Zhu et al.^24^ have discovered that XIST is OE in cervical cancer tissues and cell lines, and this overexpression is also demonstrated by another study focusing on cervical cancer^10^. Moreover, miR-149-3p expression has been demonstrated to be repressed in tumor-bearing mouse spleens in BC^25^ and Sun et al.^14^ have supported that miR-149 is decreased in OC tissues and cells. A higher expression of FOXP3 in epithelial OC tissues at advanced stages has been found in a recent study^26^. Besides, we verified that the high expression of XIST indicated a poor prognosis of OC patients and the predictive role of XIST in prognosis of BC patients has been identified as well^27^. In addition, we found through online prediction, dual luciferase reporter gene assay, and RNA pull-down assay that XIST could bind to miR-149-3p, and FOXP3 was confirmed as a direct target of miR-149-3p. Consistently, the binding relationship between XIST and miR-149-5p has been identified in osteoarthritis^16^, whereas the targeting relationship between miR-149-3p and FOXP3 remains largely unknown.

In addition, the OC cells were accordingly transfected with silenced/OE-XIST or miR-149-3p mimic/inhibitor, and the effects of altered XIST and miR-149-3p on OC cell phenotypes were observed. The outcomes reflected that reduction of XIST or elevation of miR-149-3p constrained proliferation and accelerated apoptosis of OC cells. In accordance with this finding, it has been recently reported that the knockdown of XIST decelerates proliferation and facilitates apoptosis of cervical cells (Siha and Hela cells)^24^ and a publication has suggested that the restored miR-149 suppresses malignant behaviors of OC cells^14^. We also found that the downregulated XIST elevated miR-149-3p to repress migration and invasion of OC cells. Similarly, XIST knockdown has been revealed to restrain migration and invasion of non-small cell lung cancer cells^28^, and the inhibitory role of elevated miR-149-3p in migration and invasion of bladder cancer cells has also been verified^29^. Furthermore, the impacts of altered XIST and miR-149-3p on tumor growth in vivo was observed and we found that deleted XIST and promoted miR-149-3p restrained OC development in vivo. A similar finding by Liu et al.^21^ has implied that reduction of XIST restricts thyroid cancer cell proliferation in vivo and it has also been pointed out that miR-149 mimic inhibits tumor growth of BC xenografts^30^.

In conclusion, we have found that inhibition of lncRNA XIST and upregulation of miR-149-3p repressed the malignant behaviors of OC cells, which may contribute to exploration on OC therapeutic strategies. Great efforts remain to be done to further investigate the functional mechanisms of XIST on OC development.
